# Physical properties of zinc, silver, or cerium ion doped borate glass incorporated PCL/gelatin electrospun fibers and their interaction with NG108-15 neural cells

**DOI:** 10.1007/s10856-025-06863-w

**Published:** 2025-02-10

**Authors:** Duygu Ege, Vida Khalili, Hsuan-Heng Lu, Heike Reinfelder, Dominique de Ligny, Aldo R. Boccaccini

**Affiliations:** 1 Institute of Biomedical Engineering, Bogazici University, Rasathane St., Kandilli, Istanbul Turkey; 2 Institute of Biomaterials, University of Erlangen-Nuremberg, Erlangen, Germany; 3 Department of Materials Science and Engineering, Institute of Glass and Ceramics, University of Erlangen-Nuremberg, Erlangen, Germany

## Abstract

In this study, 1393-B3 based borate bioactive glasses (BGs) undoped and doped with 1 wt% zinc (ZnBG), cerium (CeBG), or silver (AgBG) were prepared and were incorporated into gelatin/PCL (GEL/PCL) electrospun fibers for neural tissue engineering applications. Particle sizes of the prepared BGs were 3.1, 10.6, 14.6, and 3.7 µm for undoped BG, ZnBG, AgBG, and CeBG, respectively. Aligned electrospun fibers were prepared with 5 wt% of BG particles to produce 5BG/PCL/GEL, 5ZnBG/PCL/GEL, 5AgBG/PCL/GEL and 5CeBG/PCL/GEL fibers. Random 5CeBG/PCL/GEL fibers were also prepared for comparison. A rise in fiber diameter was measured for BG-incorporated fibers compared to PCL/GEL fibers. Mechanical tests on the fibers indicated ultimate tensile strength values of 1–3.5 MPa, the range of mechanical properties of neural tissue. Cell culture studies were carried out with the NG108-15 cell line. Cell alignment was observed on the electrospun fibers on day 2. On days 1 and 2, the optical density was higher for ZnBG/PCL/GEL, CeBG/PCL/GEL, and AgBG/PCL/GEL than for BG/PCL/GEL fibers. On day 4, undoped BG-containing nanofibers had higher optical density compared to those containing doped BGs. This result could be due to a slower release rate of boron from the pure BG/PCL/GEL fiber mat. Overall, within the studied range, all fiber mats were found to be suitable for neural tissue engineering in terms of neural cell compatibility and mechanical properties. In the future, a wider range of ion doping must be considered to fully comprehend the potential of such ion-releasing fibers for neural regeneration.

**Graphical Abstract:**

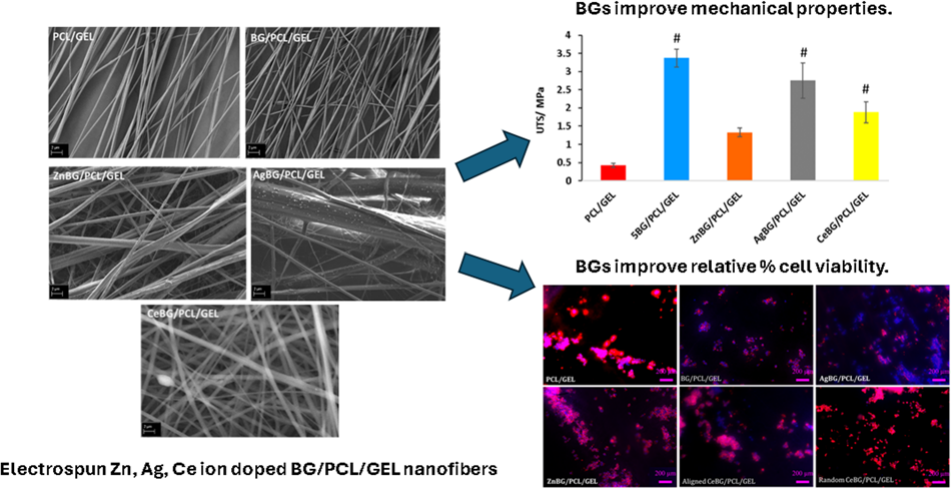

## Introduction

Electrospinning is a widespread and affordable technology for producing scaffolds for tissue engineering [1–3]. In this process, a high-intensity electric field is applied between a charged fiber collector and a needle tip coupled to a syringe pump [3–6]. Poly(caprolactone) (PCL) is a commonly studied polymer for electrospinning. It is a semi-crystalline synthetic polymer that is non-toxic, biocompatible, and biodegradable. As PCL degrades slowly in vivo, it is used when a prolonged healing time is necessary. The structural support provided by PCL allows for cell growth and proliferation in vivo [7]. On the other hand, gelatin is a biodegradable and biocompatible natural polymer derived from collagen that promotes cell adhesion and growth. Gelatin, however, exhibits weak mechanical properties. As a result, blending PCL and gelatin can achieve both desirable physical and mechanical properties for tissue engineering applications [8, 9].

Nerve regeneration represents an important potential application of aligned electrospun fibers [10]. Aligned electrospun fibers can act as neural guidance conduits for the regeneration of damaged peripheral neurons. This enables the diffusion of neurotrophic factors and axonal growth [11]. Originally developed for bone substitution and regeneration [12], PCL/gelatin fibers are also used for neural tissue engineering. For example, PCL nanofibers have been shown to enhance the differentiation of stem cells into neurons and promote neural outgrowth [13]. Various studies indicated that gelatin can be used to improve neural growth [3, 14]. For example, Farzamfar et al. [15] produced stem cell-laden PCL/gelatin conduits that were implanted in the sciatic nerve of Wistar rats. The immunophenotyping studies indicated that the PCL/gelatin conduits enhanced functional recovery of sciatic nerves similar to autografts, which is currently the gold standard for neural tissue regeneration. In another study, human induced pluripotent stem cell (hiPSC) laden PCL/gelatin electrospun nanofibers were produced [16]. Immunocytochemistry studies indicated that the PCL/gelatin nanofibers provided mechanical and biological support for the neural differentiation of hiPSC [16].

Bioactive glasses, being increasingly considered for soft tissue engineering, are also found to support peripheral neural regeneration [17–21]. Bioactive glasses release therapeutic dissolution by-products that trigger neural healing, and many bioactive glass compositions are studied to favor neural regeneration [22]. To give this an example, 30 wt% of silicate bioactive glass (45S5 composition) was incorporated in gelatin to produce neural conduits which were implanted in the sciatic nerve of Wistar rats and histological studies indicated that these scaffolds were suitable for neural regeneration [23]. Zhang et al. [22] incorporated 20 and 40 wt% of Si–Ca–Na–Zn–Ce bioactive glasses in polylactide-co-glycolide composites to produce neural guidance conduits and calcium ion release from these neural conduits had beneficial effects on neural healing. The incorporation of therapeutic ions into borate bioactive glasses (BGs) also provides interesting properties for tissue engineering applications [24]. Marquart et al. [25]. produced aligned 35 mg/ml or 70 mg/ml BG microfiber incorporated fibrin scaffolds which supported directed axonal growth of dorsal root ganglia (DRG). The cell culture studies with DRG indicated the biocompatibility of the produced scaffolds. Gupta et al. [26] produced 50 wt% BG-incorporated PCL scaffolds for neural regeneration. In the study, BGs were also produced with different dopants including 1 wt% of silver, cerium, iron, gallium, yttrium, and zinc. Then, nerve outgrowth from DRG explants was evaluated. The dopants were observed to have a positive influence on DRG nerve outgrowth in a dose-dependent manner. On day 7, zinc-doped BG/PCL increased nerve outgrowth the most, followed by iron and gallium-doped BG/PCL. In comparison, for the studied compositions, bare BG, silver, cerium, and yttrium-doped BG/PCL scaffolds had lower nerve outgrowth.

In the context of the emerging field of ionic therapeutics, biologically active ions are found to have interesting effects on neural regeneration [27–33]. Recently, cerium oxide nanoparticles attracted considerable interest because of their potential therapeutic applications. In particular, the oxygen buffering capacity of ceria is well established [27]. Deliormanli [28] reported that cerium oxide nanoparticles act as neuroprotective agents. They limit the amount of oxygen required to kill the cells. Ce may act as both network former and network modifier in BGs [34]. Additionally, Ce ion doping in BGs has been reported to promote angiogenesis [18]. Zn ion is also reported to be involved in a variety of physiological processes, including cell proliferation [35, 36]. Zn is commonly used for bone tissue engineering applications, however there are many studies reporting the significance of Zn ions in neural regeneration [29–32, 37, 38]. It is indicated that in a dose-dependent manner, Zn is essential for neurological activities; however, a high dosage of Zn may be highly toxic for neural cells and even cause Alzheimer’s disease in the long term [39]. Ag is a commonly studied ion for applications such as bone regeneration and wound healing due to its anti-bacterial properties [33]. The literature indicates that Ag may be toxic to neural cells; however, at low doses, its use leads to cytocompatibility and, additionally, Ag provides anti-bacterial activity [40–43].

In this study, undoped and Zn, Ce or Ag doped 1393B3 borate bioactive glasses (BGs) (compositions: see Table 1) were produced and incorporated in particulate form in aligned PCL/GEL fibers obtained by electrospinning. Additionally, Ce-doped 1393B3 BG particles were incorporated in random PCL/GEL fibers to study the impact of the alignment of fibers on the material mechanical and biological properties. The produced fibers were characterized in terms of physical and mechanical properties and cell culture studies were conducted with NG108-15 cells (hybrid cell line of mouse neuroblastoma cells and rat glioma cells). The potential of the produced aligned fiber mats for neural tissue engineering was assessed.

**Table 1:** The experimental groups of BGs used in this study (in wt%)

	B_2_O_3_	CaO	K_2_O	Na_2_O	MgO	P_2_O_5_	ZnO	CeO_2_	Ag_2_O
1393B3 BG	53	20	12	6	5	4	0	0	0
ZnBG	52	20	12	6	5	4	1	0	0
CeBG	52	20	12	6	5	4	0	1	0
AgBG	52	20	12	6	5	4	0	0	1

## Materials and Methods

### Synthesis of bioactive glasses

Bioactive glasses based on the borate 1393B3 composition (53B_2_O_3_–20CaO–12K_2_O–5MgO–6Na_2_O–4% P_2_O_5_ in wt %) (see Table 1 for all compositions) were produced by conventional melt-quenching technique [19]. Na, K, and Ca were introduced as carbonates, B as H_3_BO_3_, and P as ammonium dihydrogen phosphate (H_6_NO_4_P). The carbonate and oxide powders were dried before weighting. Then the mixed powders were first decarbonated before being melted twice at 1050°C and quenched by immersion of the bottom of the platinum crucible in water. Between the two melting steps, the glass was ground to ensure a better homogeneity of the final product. The measured compositions within the experimental error of few tenth of a percent do not depart from the nominal compositions. This reference batch was then remelted in 25 g units doped with 1 wt% of CeO, AgO or ZnO. Each doped sample was melted at 1000 °C, ground, and remelted to ensure homogeneity. The nominal composition of each glass sample is given in Table 1.

### Electrospinning process

To prepare solutions for electrospinning, first, 1.875 g of PCL (80 kDa, Sigma Aldrich, Germany) was dissolved in 10 ml of acetic acid (VWR, Darmstadt, Germany), and 0.375 g of gelatin was dissolved in 1.66 ml of formic acid and mixed for 1 hour. After this, the gelatin solution was added to the PCL solution. For the preparation of BG-incorporated solutions, 5 wt% BGs (bare BG, ZnBG, AgBG, CeBG) with respect to PCL was added in the PCL solution. The solutions were ultrasonicated before electrospinning.

After mixing the solution overnight, the solution was fed at 0.3 mL/h through a 23 G needle. Fibrous mats with 83.3% PCL and 16.7% gelatin (PCL/GEL) were prepared by using a commercial electrospinning equipment (EC-CLI, IME Technologies Netherlands). Nitrogen flux was set at 8 mL/min, and the temperature and relative humidity were kept at 23 °C and 40%, respectively. Then, the electrospinning process for aligned fibers was conducted by keeping the distance between the target and the needle fixed at 11 cm and the applied voltage was 15 kV at the needle and -2 kV at the rotating mandrel (1500 rpm). Additionally, for the production of random nanofibers of CeBG-PCL/GEL, a rotating mandrel was used at a speed of 500 rpm. The fiber diameter distribution was analyzed from 55 fibers using ImageJ software for all fiber bodies produced.

### Characterization studies

Scanning electron microscopy (SEM) was used to analyze the morphology of the BGs and the fiber mats using an Auriga SEM instrument (Zeiss, Oberkochen, Germany). Qualitative compositional analysis was carried out by energy-dispersive X-ray spectroscopy (EDX).

Structural analysis was performed by Fourier Transform Infrared Spectroscopy (FTIR; IRAffinity-IS, Shimadzu) in attenuated total reflectance (ATR) mode, using a wavenumber range of 4000 to 400 cm^−1^ with a resolution of 4 cm^−1^ with 32 spectral scans.

X-ray diffraction (XRD) patterns of the fiber mats were obtained using an X-ray diffractometer (Miniflex, 600, Rigaku) in the 2θ range of 10° to 80° equipped with Cu Kα radiation. A step size of 0.02° and dwell time of 1° per minute were used.

Static contact angle was measured using a drop shape analyzer DSA30 (Kruss GmbH) to determine the wettability of fiber mats.

For mechanical testing, samples were cut into rectangular shapes with 4 cm length and 5 mm width. Uniaxial tensile strength was measured using a universal testing machine (5960 Dual Column Tabletop Testing System, Instron®, Darmstadt, Germany) at room temperature. A crosshead speed of 10 mm/min using a 50 N load cell was used to carry out measurements with five replicates for each study group.

Degradation studies were carried out in phosphate buffer saline (PBS) solution for 7 days at 37 °C using scaffold holders (CellCrownTM 24, Scaffdex, Sigma Aldrich, Germany). pH measurements were taken each day for three replicates.

Inductively Coupled Plasma Optical Emission spectroscopy (ICP-OES) measurements were carried out to determine the release kinetics of ions as dissolution products of BGs from electrospun fiber mats in PBS (Perkin Elmer, Model, Avio 200). The analysis was used to determine the amount of B, Ce, Zn, and Ag ions in the PBS solution after two days of incubation of the electrospun fiber mats.

### Biological tests

NG108-15 cells (a hybrid cell line of mouse neuroblastoma cells and rat glioma cells) (Sigma) were used to investigate the % cell viability and cell morphology. Disk-shaped electrospun mats were placed in sample holders (Scaffdex, CellcrownTM, Sigma Aldrich, Munich, Germany). The samples were placed in 24-well plates and the samples were disinfected by exporuse to UV light for 1 hour before seeding cells. WST-8 assay (CCK-8, Sigma) was carried out on days 1, 2 and 4 after seeding the cells. The cells were also stained with 8 µL/mL rhodamine phalloidin and 1 µL/mL DAPI (Thermofisher Scientific) and imaged by a fluorescence microscope (Axio Scope A1, Zeiss, Jena, Germany).

## Results

**Fig. 1:**
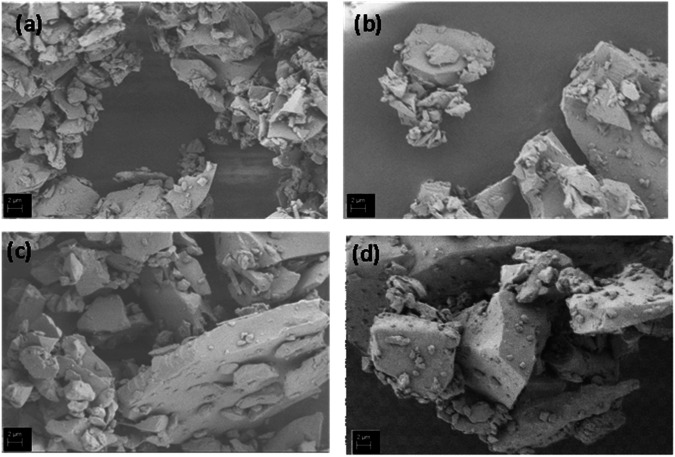
SEM images of (**a**) pure BG, (**b**) AgBG, (**c**) ZnBG, (**d**) CeBG microparticles, showing their irregular morphology

Figure 1 shows SEM images of the microparticles.

**Fig. 2:**
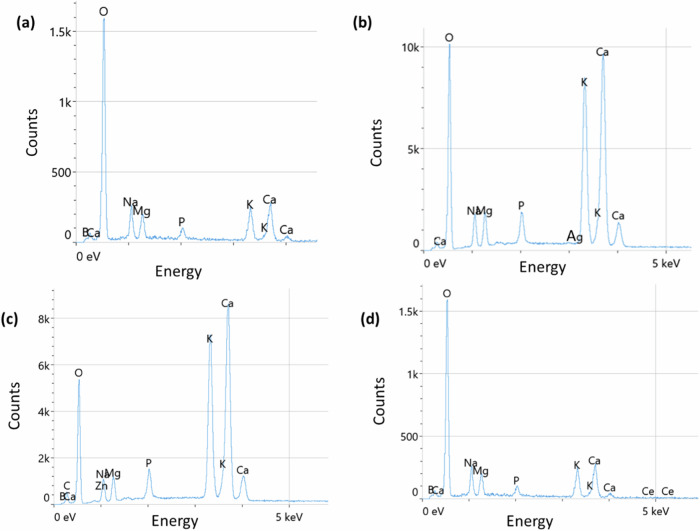
EDX compositional analysis of (**a**) BG (**b**) AgBG, (**c**) ZnBG, (**d**) CeBG microparticles

Figure 1 shows that all the microparticles have an angular and irregular geometry. Figure 2 illustrates EDX compositional analysis of all prepared BGs which proves the successful ion doping.

**Fig. 3:**
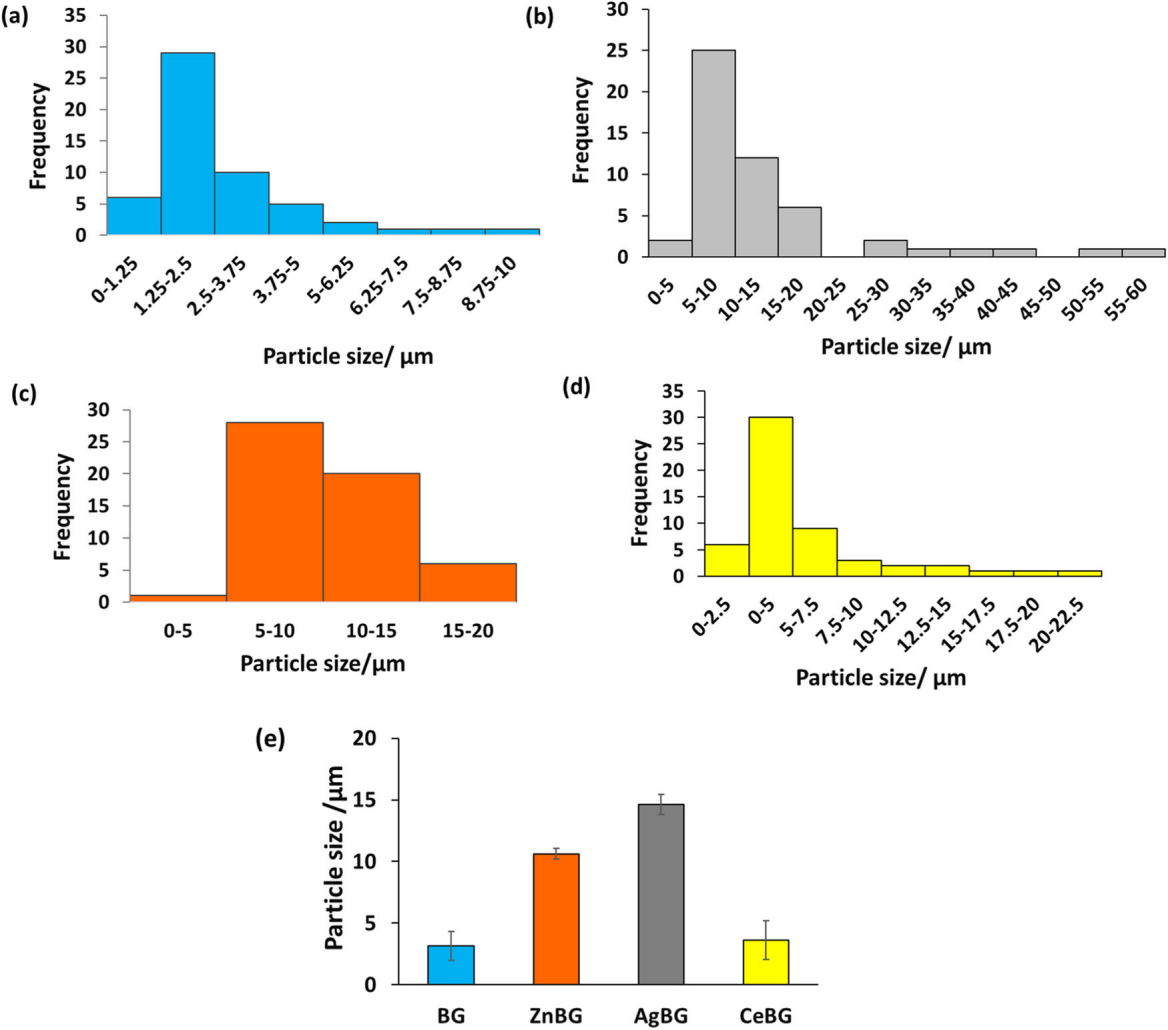
Particle size distribution of (**a**) BG, (**b**) AgBG, (**c**) ZnBG, (**d**) CeBG microparticles and (**d**) mean particle size of the BGs in comparison (*n* = 55)

Figure 2 proves that all ions were successfully doped into the relevant BGs. All spectra show Ca, Na, Mg, P, and K ion peaks which are the relevant peaks for 1393B3 BG. For AgBG, ZnBG, and CeBG samples, peaks corresponding to Ag, Zn, and Ce can be observed, respectively. Figure 3 shows the particle size distribution of the produced BG microparticles.

**Fig. 4:**
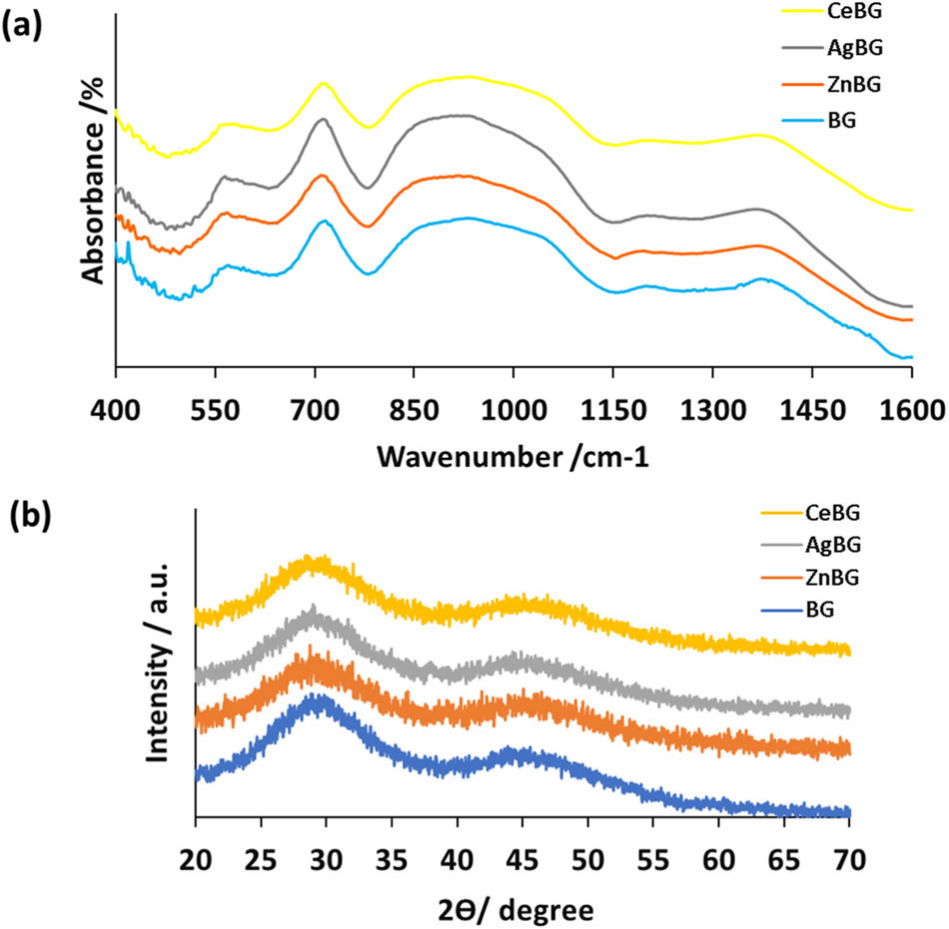
(**a**) FTIR spectra and (**b**) XRD patterns of BG microparticles used in this work

Figure 3 shows the unimodal particle size distribution of the BG particles in all compositions. The size distribution is found to be quite wide, especially for AgBG microparticles. A higher particle size was measured for Zn and Ag-doped particles. Figure 4 presents FTIR spectra and XRD patterns of the different BGs produced.

**Fig. 5:**
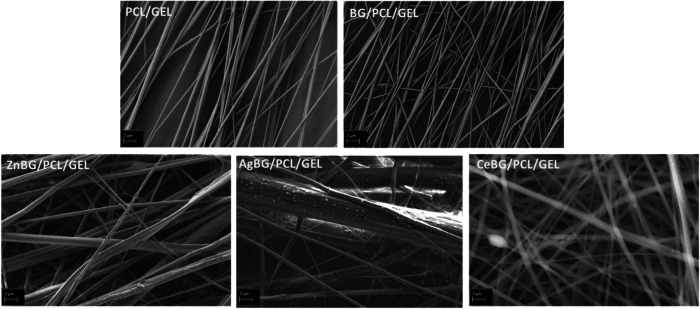
SEM micrographs of the produced fiber mats

In Fig. 4a, the resonance at around 720 cm^−1^ is due to the bending mode of the BO_3_ group [44]. The resonance at 1370 cm^−1^ is for the B-O stretching mode of the BO_3_ group [44]. The resonance at 970 cm^−1^ is assigned to the B-O stretching mode of the BO_4_ group [18, 44]. The resonances were the same after Ce, Ag, and Zn ion substitution indicating the sustained integrity of the borate structure after ion doping. Moreover, XRD patterns demonstrate the amorphous structure of all BGs. Thus, incorporation of Zn, Ag, or Ce ions did not cause crystallization of the glass structure [18, 44, 45]. Figure 5 shows SEM images of the electrospun fiber mats for the different study groups.

**Fig. 6:**
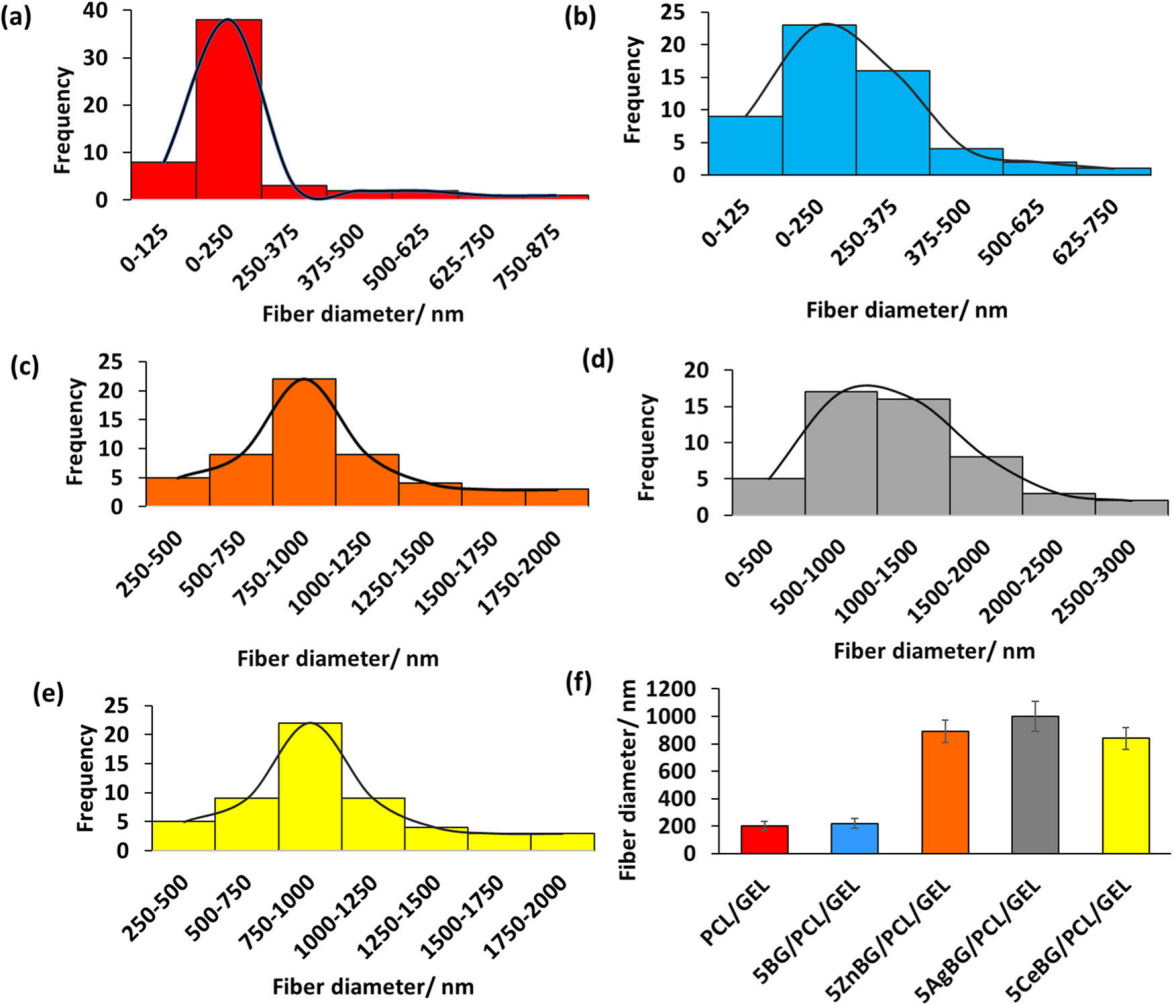
Fiber size distribution of the produced fiber mats: (**a**) PCL/GEL, (**b**) 5BG/PCL/GEL, (**c**) 5ZnBG/PCL/GEL, (**d**) 5AgBG/PCL/GEL (**e**) 5CeBG/PCL/GEL fiber mats and (**f**) peak fiber diameter of the produced fiber mats (*n* = 55)

Figure 5 shows that all produced fibers have a high degree of alignment. The degree of alignment is observed to be slightly lower for 5CeBG/PCL/GEL fibers. It is also apparent that the fiber sizes are in the same range for different fiber mats, however, qualitatively, different fiber diameters are observed. For CeBG/PCL/GEL, also random fiber mats were produced. Figure S1 shows random fiber orientation for random CeBG/PCL/GEL fiber mats. These aligned and random fiber mats were compared afterward for cell biological studies, as shown further below. Furthermore, Figure S2 shows that the fibers appear aligned even at wide-field SEM images which confirms the aligned arrangement of the fiber mats for all compositions. Moreover, as shown in Figure S3, calcium ion was distributed evenly within the fiber mat, which indicated the homogeneous distribution of the BG microparticles within the fiber mats. Figure 6 shows the fiber diameter range of the produced scaffolds.

**Fig. 7:**
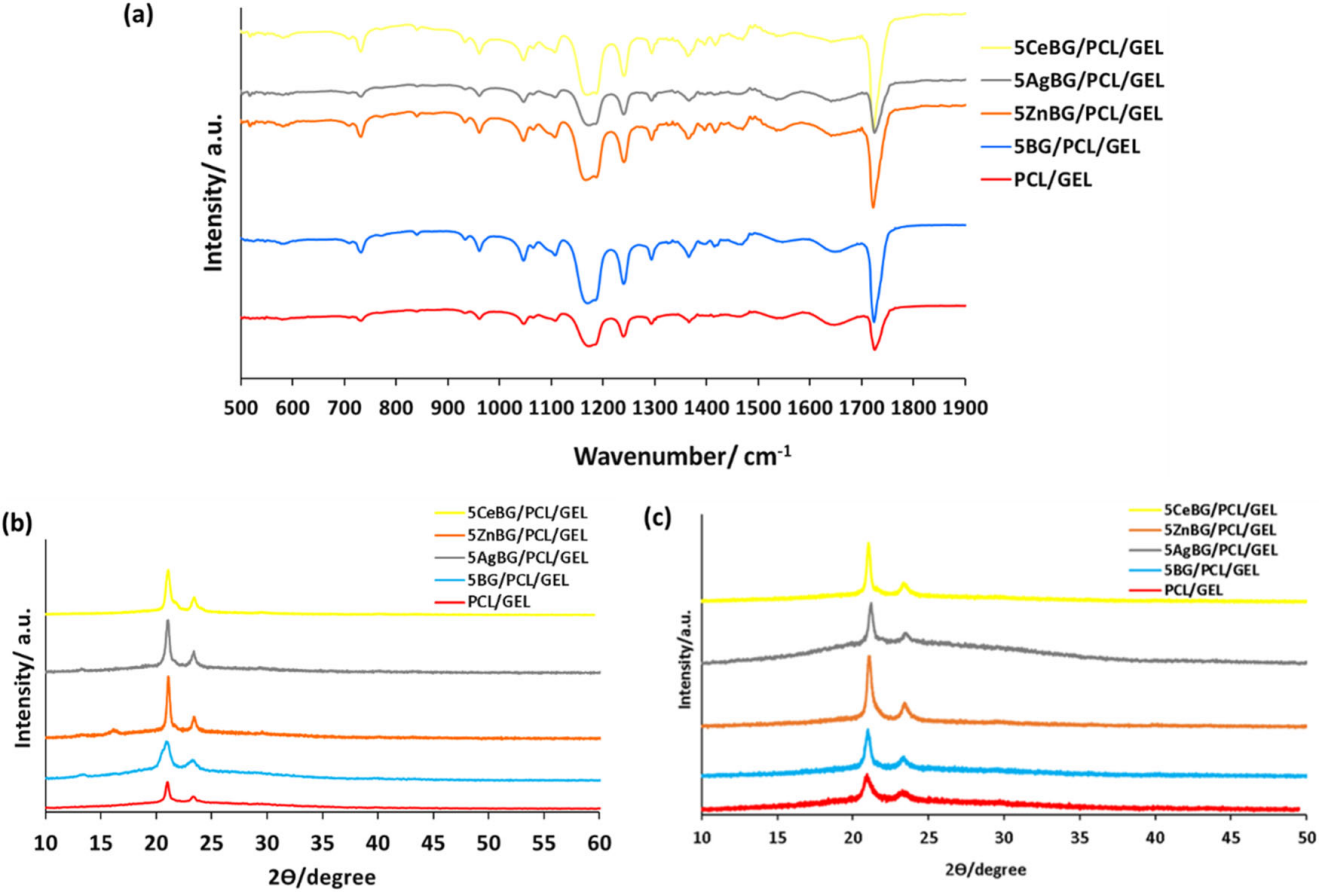
**a** FTIR spectra and (**b**) XRD patterns of PCL/GEL fiber mats, (**c**) XRD patterns of the nanofibers after SBF treatment for 7 days (the different FTIR bands and XRD peaks are explained in the text)

Figure 6 indicates that the fiber size increased significantly for Zn, Ag, and Ce-doped BG incorporated fibers. The fiber size was about 200 nm for PCL/GEL and 5BG/PCL/GEL nanofibers. With the incorporation of the Zn, Ag and Ce ions, the fiber diameter increased up to 1 micron. Figure 7 shows characteristic FTIR spectra and XRD patterns of the fibers.

**Fig. 8:**
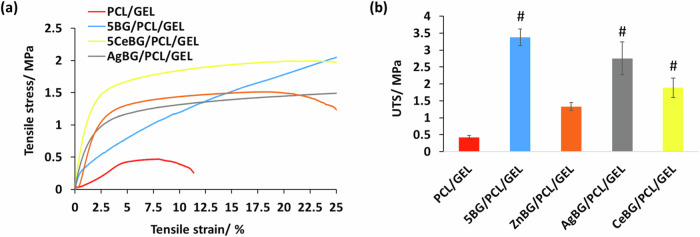
Mechanical analysis: (**a**) stress-strain curves of fiber mats, (**b**) ultimate tensile strength (UTS) of fiber mats (*n* = 5) (#*p* < 0.05 in comparison to the PCL/GEL study group)

In Fig. 7a, FTIR spectra demonstrate characteristic peaks for the fibers. PCL peaks found at 1727, 1240, and 1169 cm^−1^ are related to carbonyl stretching, asymmetric and symmetric stretching of C-O-C bonds, respectively [46]. GEL peaks were at 1651 cm^−1^ and 1514 cm^−1^ for amide I and N-H deformation of amide II, respectively [19, 47]. BG typical peaks are not detectable due to the low amount of BG microparticles in the composite [48, 49]. Figure 7b shows PCL/GEL fibers as well as BG-incorporated fibers. The two sharp peaks at 2ϴ = 21.3° and 23.6° belong to PCL. Gelatin and BG particles, due to their amorphous nature, showed no peaks [50]. Figure 7c shows the XRD patterns of the nanofibers after treatment with SBF for 7 days, showing no indication of the formation of apatite. Figure 8 illustrates the mechanical test results on the fibers.

**Fig. 9:**
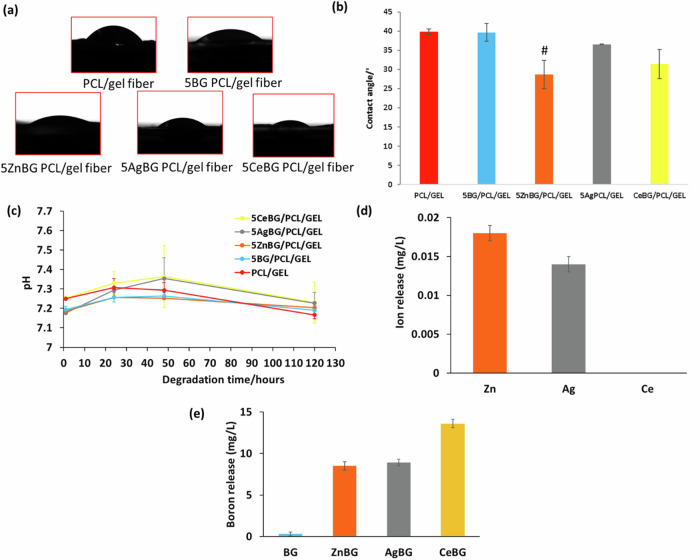
Results of (**a**, **b**) contact angle measurements, (**c**) pH during degradation studies (*n* = 5), (**d**) release of the substituted ion from the fiber mats, and (**e**) boron ion release after 48 hours in PBS (*n* = 3) (#*p* < 0.05 in comparison to the PCL/GEL study group)

The results indicate that the ultimate tensile strength of the fibers increased gradually with the addition of all types of BG microparticles. The tensile strength values were in the range 0.5–3.5 MPa. Additionally, although not significantly different, non-doped BG-incorporated fiber mats had higher ultimate tensile strength than the other study groups. Figure 9 shows the contact angle measurement, pH, and ICP ion release results on the different nanofibers.

Figure 9a and b show that the contact angle values were similar for all scaffolds, ranging from 30° to 40°. Figure 9c indicates that the peak value of pH occurs later for CeBG/PCL/GEL and AgBG/PCL/GEL fibers than for the other study groups. However, there was no significant difference between the pH levels of each biomaterial; but there is a rise of pH over time. Figure 9d illustrates Zn and Ag ion release from the fiber mats on day 2, whereas Ce ion release was not observed. However, the concentration of released ions was found to be very low. Figure 9e demonstrates that boron release from BG/PCL/GEL was lower than from the ion-doped groups on day 2.

**Fig. 10:**
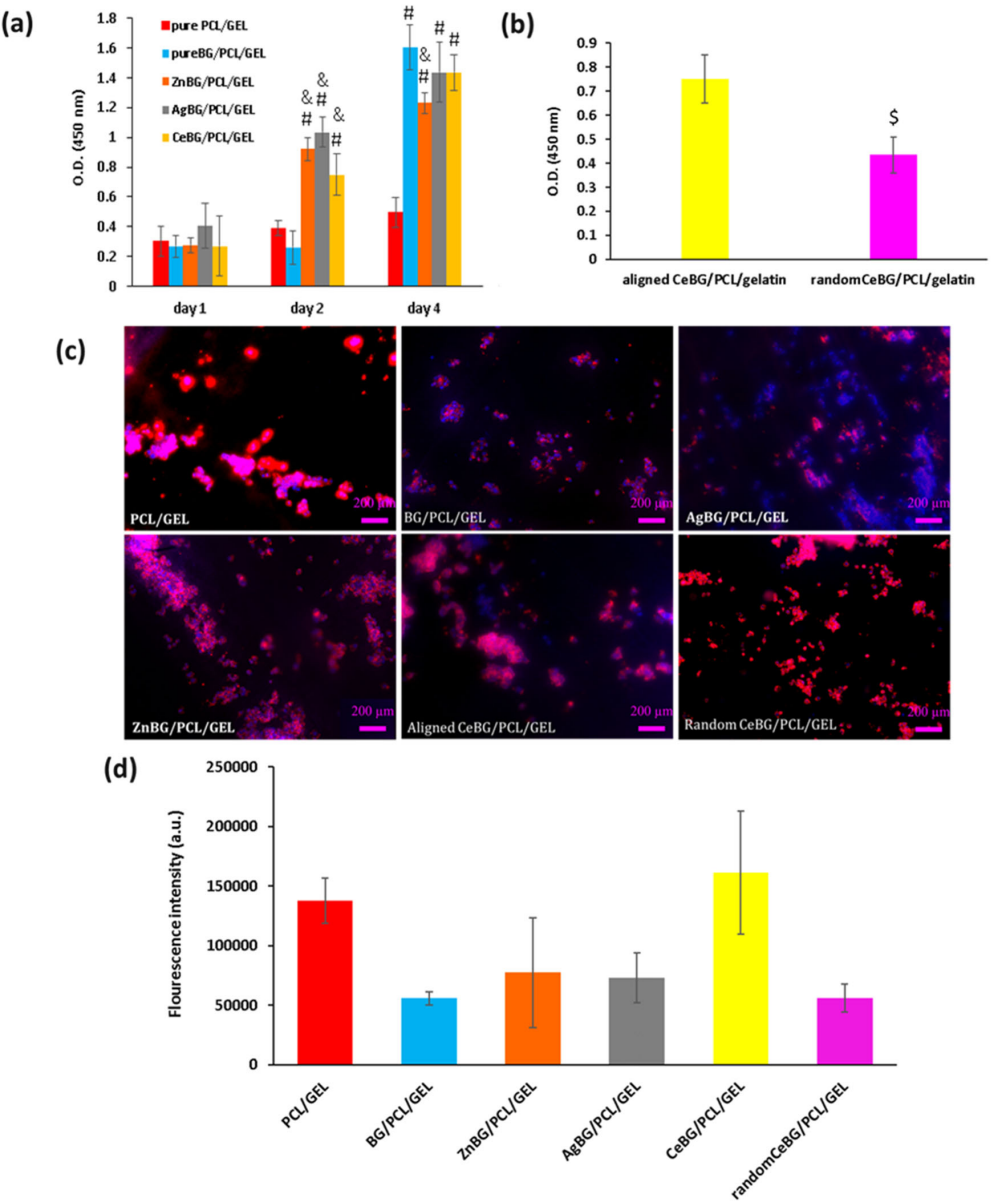
Results of cell culture study: (**a**) optical density (O.D.) of NG108-15 cells on electrospun fibers, (**b**) optical density of NG108-15 cells on aligned and random CeBG/PCL/GEL fibers, (**c**) fluorescence microscopy images of NG108-15 cells on different electrospun fibers after staining F-actin with phalloidin red and nuclei with DAPI after 2 days (scale bar = 200 μm), (**d**) flourescence intensity on day 2 (*n* = 6) (#*p* < 0.05 in comparison to the PCL/GEL study group, & *p* < 0.05 in comparison to the BG/PCL/GEL study group and $ *p* < 0.05 in comparison to aligned CeBG/PCL/GEL study group)

Figure 10 shows the results of the cell culture studies carried out on the fiber mats. Figure 10a shows that on day 2, ion doped BG incorporated fiber mats increased optical density. On day 4, the % cell viability was significantly higher for 5BG/PCL/GEL, ZnBG/PCL/GEL, AgBG/PCL/GEL and CeBG/PCL/GEL in comparison to PCL/GEL fibers. Indeed, on day 4, ZnBG/PCL/GEL fibers had significantly lower optical density than BG/PCL/GEL fibers. Moreover, as shown in Fig. 10b, aligned CeBG/PCL/GEL fibers had higher optical density than random CeBG/PCL/GEL fibers. In Fig. 10c, fluorescence intensity showed no significant difference among the different study groups; however, aligned CeBG/PCL/GEL fibers led to insignificantly higher fluorescence intensity on day 2. Figures S4, S5 and S6 show that the fluorescence intensity had no significant difference between study groups on day 4 while ZnBG/PCL/GEL fibers had nonsignificant higher fluorescence intensity.

## Discussion

In this study, five different types of aligned PCL/GEL fiber mats were produced with pure undoped borate BG, Zn incorporated BG, Ag incorporated BG and Ce incorporated BG microparticles. Random oriented 5CeBG/PCL/GEL fibers were also produced for comparison purposes.

Firstly, although an unimodal particle size distribution was achieved for all the microparticles, the size distribution was found to be quite wide, especially for AgBG microparticles. A wide-size distribution of BG microparticles is also reported in the literature [28]. In our study, Ag and Zn led to an increase in microparticle size. Literature shows that addition of both Ag and Zn can increase the mechanical properties of bioactive glasses [51–53].

Figure 6 indicated a significant increase in fiber size for fibers containing Zn, Ag, and Ce doped BG. This may be due to changes of solutions’ electrical conductivity, surface tension, and viscosity due to incorporation of different types of BGs [54–57]. High rotation speed of the collector, leads to smaller fibers and enhanced alignment [58]. Probably, due to factors such as, conductivity, surface tension and viscosity, non-doped BG-incorporated fiber mats had both smaller fiber diameters and enhanced alignment than the other groups [58, 59].

The mechanical properties of scaffolds should be suitable to support cells during attachment and proliferation, therefore it is important to match the mechanical properties of the native tissue to achieve improved cell attachment and proliferation [7]. The findings suggest that the ultimate tensile strength of the fibers increased with the incorporation of all types of BG microparticles. The tensile strength values were between 0.5 to 3.5 MPa, aligning with the tensile strength of neural tissue [60]. Nevertheless, although the differences were not statistically significant, the fiber mats with non-doped BG showed higher ultimate tensile strength compared to the other groups in the study. This could be due to better alignment of the fibers in this study group in comparison to the other scaffolds [19].

Cell behavior is strongly affected by surface wettability and the optimal water contact angle is reported to be between 40 and 70°, considering that high hydrophilicity or hydrophobicity is not favorable for cell adhesion [61]. The contact angle values were found to be between 30° and 40° which indicated that it was in the lower range of the optimal contact angle range for tissue engineering applications [61]. Moreover, pH measurements indicated that all samples were at the physiological pH of tissue; therefore, no toxic effects are expected from the scaffolds due to pH variations. ICP study was conducted to assess the ion release from the scaffolds. Zn and Ag ions were released from the fiber mats on day 2, but no release of Ce ions was observed. Nevertheless, the concentration of the released ions was found to be very low, being lower than the effective physiological dosages which are approximately 1 µM for Zn and Ag ions [18]. Boron release from BG/PCL/GEL was lower than from the ion-doped groups on day 2. This is likely because Zn, Ag, and Ce can act as network modifiers which should lead to faster release of boron ions due to modification of the glass structure [18, 34].

Optical density measurements of the samples were taken after cell culture studies for 4 days. The increase of optical density values for different groups over time indicated an increase of % cell viability on the prepared sample groups. The significant increase of optical density for 5BG/PCL/GEL fibers from day 2 to 4 could be due to the slower dissolution rate of boron ions from 5BG/PCL/GEL than for the other study groups until day 2, which was compensated on day 4. Indeed, on day 4, ZnBG/PCL/GEL fibers had significantly lower optical density than BG/PCL/GEL fibers. The different optical density of the aligned CeBG/PCL/GEL fibers and the random CeBG/PCL/GEL fibers is possibly because of the higher positive effect on cells exerted by aligned fibers in comparison to random fibers [14]. Moreover, insignificantly higher fluorescence intensity for aligned CeBG/PCL/GEL may indicate further actin polymerization on aligned CeBG/PCL/GEL fiber mats in comparison to the other scaffolds [62]. Literature indicates that CeBG improves the cytocompatibility of bone marrow-derived stem cells; however, this occurred at about 10 wt% doping of Ce ions, which is reported to occur due to neutralization of oxidative stresses in the presence of Ce ions [63]. Therefore, it is reasonable to study BGs with higher Ce doping concentrations to investigate the effect of this ion on neural cells.

On the other hand, Ag ions have been reported to reduce cytocompatibility above 2 wt% doping in BGs [64]. Since in this study the Ag concentration is below the mentioned toxic dosage, cells showed no cytotoxicity. In the literature, it is reported that doping of 0.6 mol% Ag ions had higher % cell viability compared to BGs with no doping, both for keratinocytes and fibroblasts [33]. A similar trend was observed in this study on day 2. On day 4, optical density was notably reduced for ZnBG-incorporated BG/PCL/GEL fibers compared for PCL/GEL fiber mats. Similarly, literature indicated that mesoporous bioactive glasses with Zn ion incorporation above 2 mg/ml significantly reduced % cell viability [65]. In the future, lower concentrations of Zn-doped BG/PCL/GEL fibers need to be studied to characterize their effect on the cytocompatibility of neural cells. Moreover, higher Ce ion concentrations should be also studied to assess the potential of CeBG-doped nanofibers for neural regeneration. On the other hand, Zn ions can be doped at lower wt% (<1 wt %) in BGs, which may trigger neural cell proliferation, as indicated in the literature [35]. Furthermore, it will be beneficial to study ion release for more extended periods to fully comprehend the effect of ion release on the cytocompatibility of the produced fibers. Moreover, in this study cells were not induced to differentiate. In the future, axonal growth should be monitored to further analyze electrospun fibers effect on neural regeneration. Additionally, porosity measurements are required to determine the effect of porosity on % cell viability measurements. Furthermore, in vivo studies will be necessary to fully assess the potential of the produced fibers for neural tissue engineering applications.

## Conclusions

In this study, undoped and Zn, Ce, Ag doped 1393B3 BG microparticles were prepared and incorporated in PCL/GEL electrospun fibers for possible use in neural tissue engineering applications. The presence of ion-doped BG particles increased the fiber size compared to undoped nanofibers or BG-incorporated nanofibers. The produced fiber mats had mechanical properties suitable for neural regeneration. Cell culture studies indicated that all fibers were cytocompatible. BG-incorporated fibers had significantly higher optical density on days 2 and 4 than PCL/GEL fiber mats. On day 4, only 5ZnBG/PCL/GEL had significantly lower optical density than 5BG/PCL/GEL, which may be due to a relatively high dose of Zn. In the future, lower Zn ion doping must be studied which may lead to higher cell proliferation. Ce ion doping also showed potential for neural regeneration and higher concentrations of Ce ions need to be studied to understand the full potential of CeBG/PCL/GEL fiber mats for neural regeneration. By further adjustment of the ion concentration, the produced fiber mats should be tested as efficient platforms for neural tissue engineering. Overall, the proposed aligned BG/PCL/GEL fiber mats with ion-releasing capability show potential for neural tissue regeneration applications and they can be considered within the emerging group of bioactive materials for ionic medicine approaches.

## Supplementary information

Supplementary file
