# Supplementary material for: Physical properties of zinc, silver, or cerium ion doped borate glass incorporated PCL/gelatin electrospun fibers and their interaction with NG108-15 neural cells
Source: J Mater Sci Mater Med. 2025 Feb 10;36(1):20. doi: 10.1007/s10856-025-06863-w (PMC11811259; doi:10.1007/s10856-025-06863-w)
Supplement: Supplementary file 1 — Supplementary file [file 10856_2025_6863_MOESM1_ESM.docx]

**Supplementary file**

Figure S1 shows aligned and random CeBG/PCL/GEL fibers.


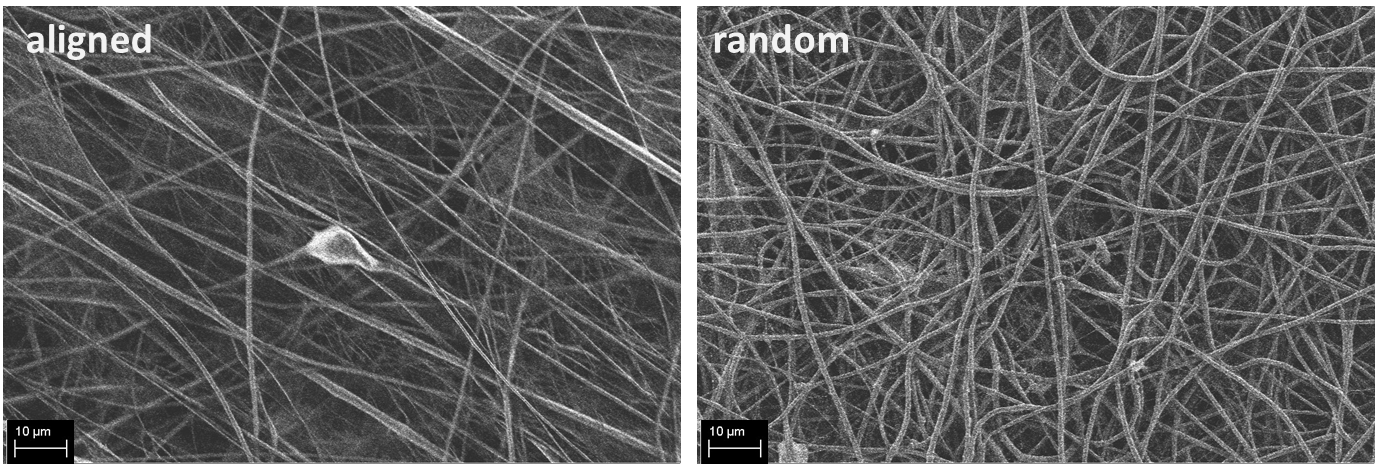


**Figure S1.** SEM image of aligned and random CeBG/PCL/gelatin nanofibers

**Table S1.** Fiber diameter of the electrospun fiber mats

| **Study group** | **Fiber diameter (nm)** |
| --- | --- |
| **PCL/GEL** | 200 ± 32 |
| **5BG/PCL/GEL** | 220 ± 35 |
| **5ZnBG/PCL/GEL** | 890 ± 83 |
| **5AgBG/PCL/GEL** | 1000 ± 107 |
| **5CeBG/PCL/GEL** | 840 ± 80 |
| **Random 5CeBG/PCL/GEL** | 1200 ± 87 |

Figure S2 shows wide field SEM images of the electrospun fibers.


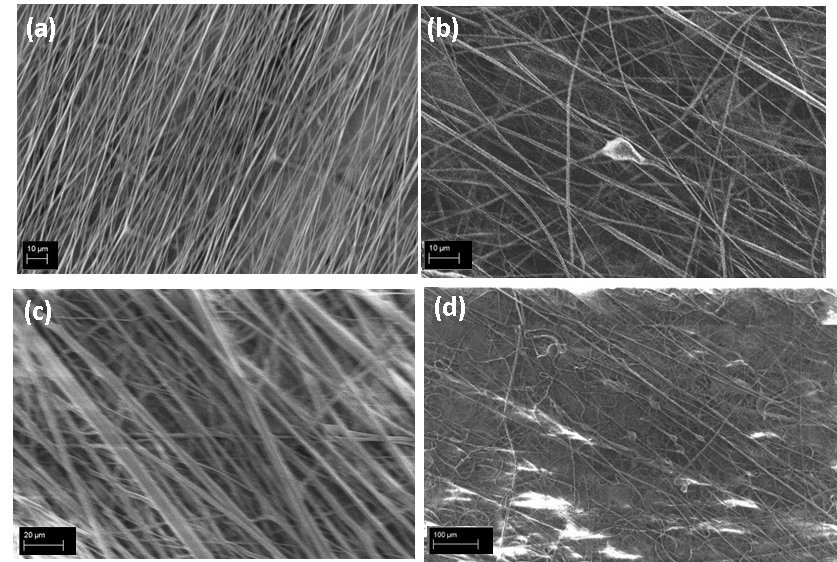


**Figure S2.** SEM image showing aligned fibers even at wide field imaging

Figure S2 illustrates quite aligned arrangement of the fibers even at wide field SEM images. Figure S3 shows the even distribution of calcium and carbon ions on the fiber mats.


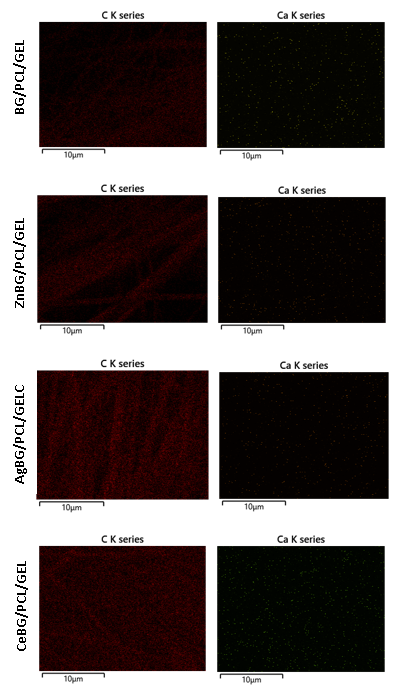


**Figure S3.** Distribution of carbon and calcium ions on the fiber mats

Figure S4 shows fluorescence microscopy images for NG108-15 cells for 1 day.


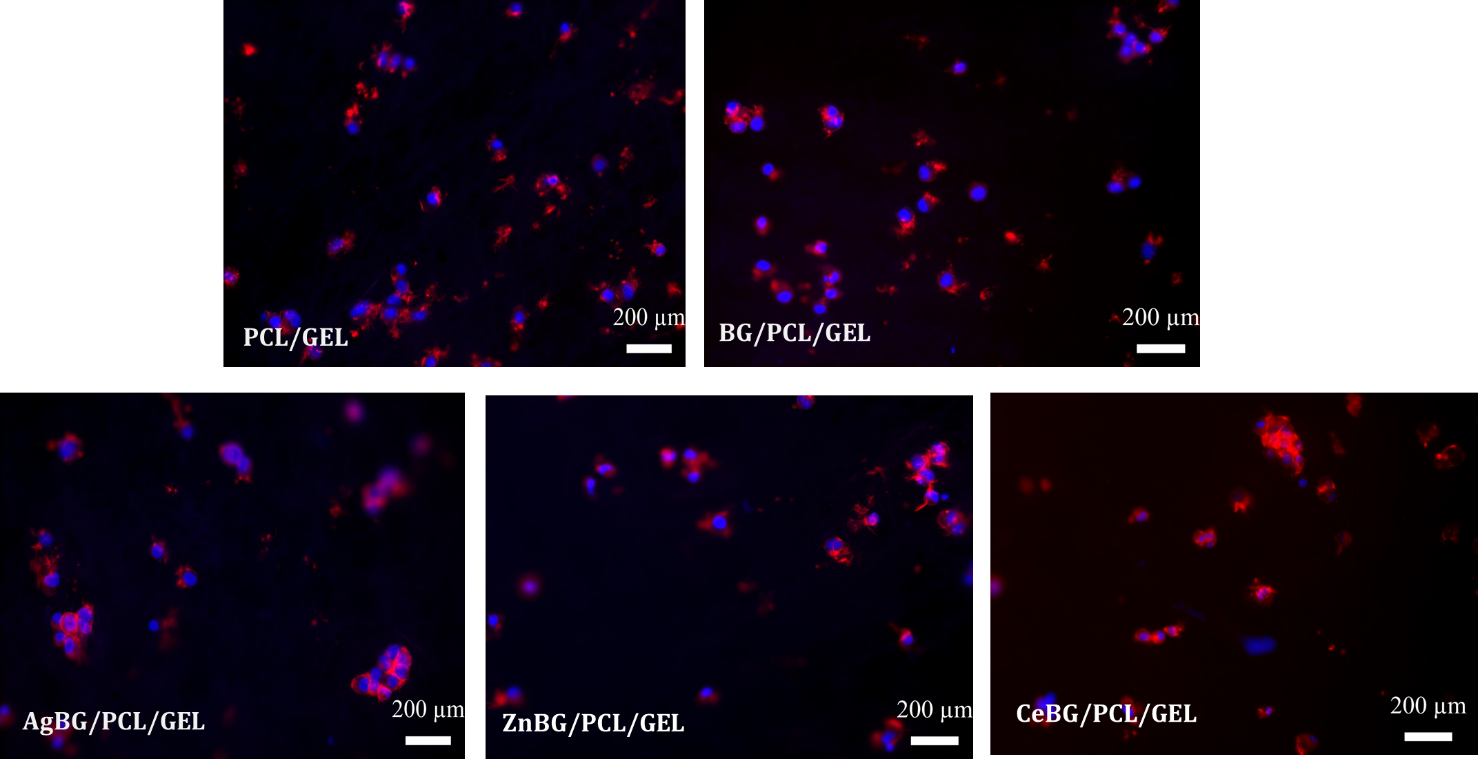


**Figure S4.** Cell culture studies (a) Optical density of the NG108-15 cells on electrospun nanofibers. Fluorescence microscopy images of NG108-15 cells on different electrospun fibers after staining F-actin with phalloidin red and nuclei with DAPI after 1 days (scale bar = 200 μm).

Figure S5 shows fluorescence microscopy images for NG108-15 cells for 1 day.


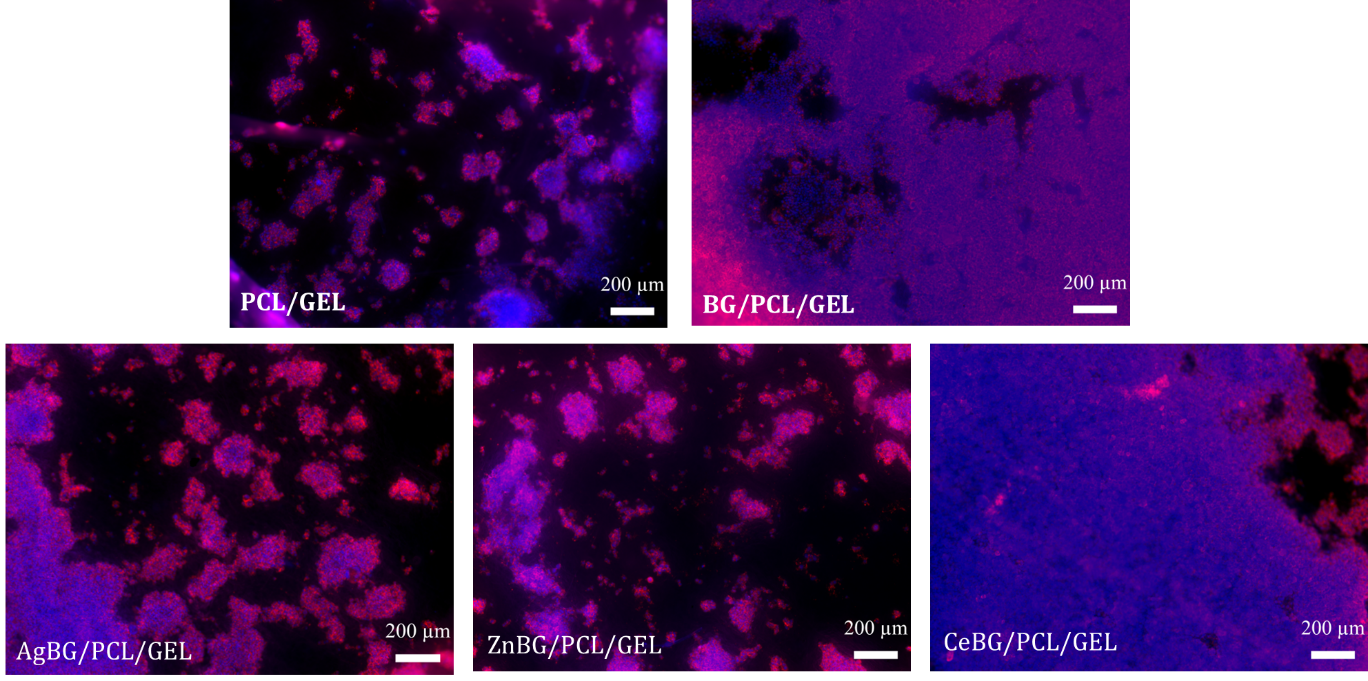


**Figure S5.** Cell culture studies (a) optical density of the NG108-15 cells on electrospun nanofibers. Fluorescence microscopy images of NG108-15 cells on different electrospun fibers after staining F-actin with phalloidin red and nuclei with DAPI after 4 days (scale bar = 200 μm).

Figure S6 shows fluorescence intensity of the study groups on day 4.


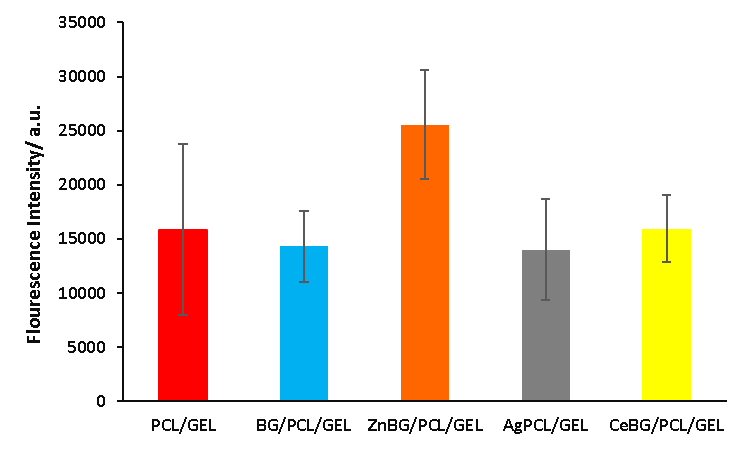


**Figure S6.** Fluorescence intensity of the study groups on day 4
